# In Silico Investigations into the Selectivity of Psychoactive and New Psychoactive Substances in Monoamine Transporters

**DOI:** 10.1021/acsomega.2c02714

**Published:** 2022-10-21

**Authors:** Michelle J. Botha, Stewart B. Kirton

**Affiliations:** Department of Clinical, Pharmaceutical and Biological Science, University of Hertfordshire, Hatfield, HertfordshireAL10 9AB, United Kingdom

## Abstract

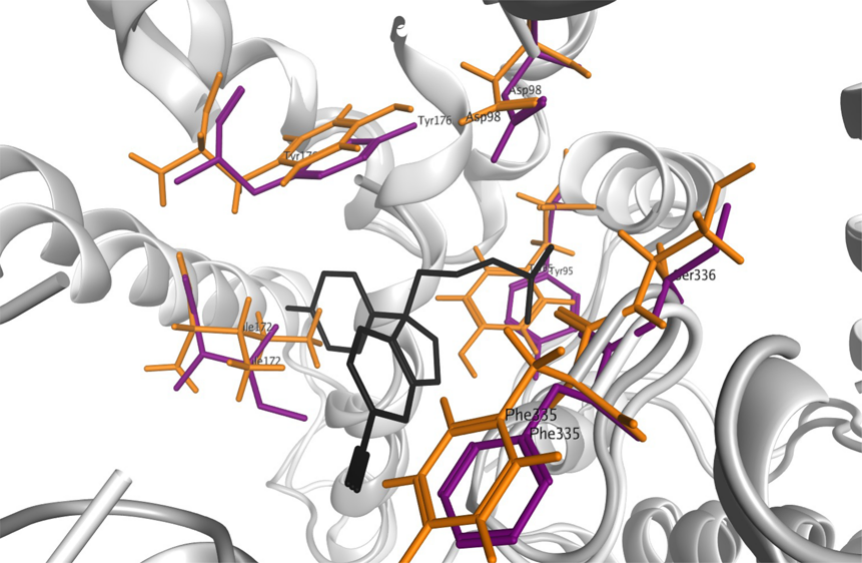

New psychoactive substances (NPS) are a group of compounds that mimic the effects of illicit substances. A range of NPS have been shown to interact with the three main classes of monoamine transporters (DAT, NET, and SERT) to differing extents, but it is unclear why these differences arise. To aid in understanding the differences in affinity between the classes of monoamine transporters, several in silico experiments were conducted. Docking experiments showed there was no direct correlation between a range of scoring functions and experimental activity, but Spearman ranking analysis showed a significant correlation (α = 0.1) for DAT, with the affinity Δ*G* (0.42), αHB (0.40), GoldScore (0.40), and PLP (0.41) scoring functions, and for DAT (0.38) and SERT (0.40) using a consensus scoring approach. Qualitative structure–activity relationship (QSAR) experiments resulted in the generation of robust and predictive three-descriptor models for SERT (*r*^2^ = 0.87, *q*^2^ = 0.8, and test set *r*^2^ = 0.74) and DAT (*r*^2^ = 0.68, *q*^2^ = 0.51, test set *r*^2^ = 0.63). Both QSAR models described similar characteristics for binding, i.e., rigid hydrophobic molecules with a biogenic amine moiety, and were not sufficient to facilitate a deeper understanding of differences in affinity between the monoamine transporters. This contextualizes the observed promiscuity for NPS between the isoforms and highlights the difficulty in the design and development of compounds that are isoform-selective.

## Introduction

Monoamine transporters (MATs) are a group of transmembrane proteins involved in regulating the concentrations of extracellular monoamine neurotransmitters, i.e., dopamine, norepinephrine, and serotonin, and as such play critical roles in the reuptake of monoamine neurotransmitters and the homeostatic regulation of presynaptic function. In terms of structure, MATs are a polytopic family of proteins in which each isoform consists of 12 transmembrane domains. The isoforms demonstrate a significant degree of homology, with areas of greatest similarity between the isoforms evident in the transmembrane domains. This high degree of conservation between the classes of MATs is lower at the N and C termini of the proteins.

The blockade of the reuptake of neurotransmitters by MATs in conjunction with the blockade of neurotransmitter receptors such as histamine H_1_, muscarinic acetylcholine, and α_1_ adrenergic receptors and the inhibition of the mitochondrial enzyme monoamine oxidase are all of interest in the development of antidepressant therapies.^1^ There are three main classes for MATs: DAT, which is responsible for the regulation of dopamine; NET, which regulates norepinephrine; and SERT, which controls the levels of serotonin.^2−6^ The reuptake of dopamine in DAT is achieved by the sequential binding and cotransport of two Na^+^ ions and one Cl^–^ ion, whereas NET and SERT uptakes of noradrenaline and serotonin, respectively, involve the sequential binding and cotransport of a single Na^+^ ion and a single Cl^–^ ion. Regulation of transporter activity at the post-translational level in all MATs is achieved through modifications like phosphorylation and N-linked glycosylation.

An appropriate balance of neurotransmitters is vital for normal brain function, as evidenced using MAT gene knockout studies in mice. As such, the role that the MATs play in the regulation of neurotransmitters is of critical importance. This means that many MAT genes have received attention for their role in the development and progression of psychiatric and neurological disorders. In addition, the identification of DAT as the neurological receptor for cocaine has provided insight into the mechanisms of addictive processes. Unsurprisingly, this means that DAT, NET, and SERT are established targets for substances that influence neurological function, including stimulants, neurotoxins, antidepressant medications, and emergent new psychoactive substances, or legal highs.

New psychoactive substances (NPS) are a collection of substances that impact neurological function similar to unlawful substances such as cocaine.^7^ Hence, given the similarities in chemical structure between known psychoactive compounds and many NPS, it is unsurprising that NPS will demonstrate affinity with the three classes of MAT^7−13^ and, due to the high degree of similarity, that ligand promiscuity is observed in the binding of NPS to DAT, NET, and SERT.^4,14−16^ A lot of research has been conducted in an attempt to develop pharmaceutical agents that target a single MAT class, such as selective serotonin reuptake inhibitors (SSRIs).^14,16−21^

It is of interest to explore molecular interactions between MATs and NPS. Such experiments have the potential to offer insight into how selectivity between the MATs could be obtained, which could then be exploited in the design of therapeutic agents. Such understanding of potential protein–ligand interactions can be achieved using a computational structure-based approach, e.g., molecular docking.

However, powerful, structure-based approaches are not without their limitations and where possible should be supplemented with indirect (or ligand-based) studies.^22,23^ Ligand-based approaches can be used to identify patterns in databases of biologically active compounds critical to imparting biological activity. If the databases are sufficiently large and there is a high level of confidence in the accuracy of the experimental activities reported for the compounds, it may even be possible to generate a predictive model of potential biological activity from the physicochemical descriptors identified as being crucial to conveying biological activity.^22,24^ Quantitative structure–activity relationship (QSAR) modeling is a technique routinely used to achieve this.^25^

The construction of robust and predictive QSAR models is reliant on high-quality experimental data. It is preferable that “self-consistent” data, i.e. data collected using the same assay under the same conditions (potentially in the same research group), are used in the construction, validation, and testing of these models. Using self-consistent data reduces the likelihood of introducing errors due to variations between laboratories. To this end, a self-consistent data set that established p*K*_i_ values for a range of psychoactive substances across the three MATs^26^ was used as the basis for these studies

This research seeks to answer the following question: can in silico methodologies be used to provide insight into why observed differences in experimental activity arise between the MAT isoforms for a series of psychoactive compounds? This study had multiple aims. The first was to establish if MAT homology models and experimental crystal structures could be used in conjunction with molecular docking methodologies and a database of known active compounds to demonstrate why differences in affinity between different NPS arise for DAT, NET, and SERT. This would facilitate the understanding of what gives rise to differences in biological activity between the MATs and what, if any, differences are observed when using high-quality comparative models compared to experimental structures. The secondary objective was to build robust and predictive QSAR models for each of the MAT classes to complement the docking studies and identify the physicochemical properties responsible for imparting the selectivity in NPS for one class of MAT over another.

## Methods

### Identification and Validation of Protein Structures

Comparative models of DAT (Q01959), NET (P23975), and SERT (P31645) were downloaded (https://swissmodel.expasy.org/repository?query=Sodium-dependent+noradrenaline+transporter), and structures were validated using Rampage,^27^ ERRAT,^28^ and Verify3D^29^ (https://saves.mbi.ucla.edu/)

Amino acid residues that violated at least one of the validation methodologies were documented. As all models were derived from the same template (PDB accession code 4M48), benchmark values for each of the tests were generated for this structure to ensure that models were not overfitted.

### Data Set for New Psychoactive Substances

The data set used in these investigations was detailed by Iversen and co-workers^26^ and contained 31 NPS with p*K*_i_ values for each of the MATs under consideration (Tables S1 and S2).

### Docking Studies

#### Putative Binding Site Identification

Binding sites for DAT, NET, and SERT were identified using SiteFinder in MOE^30^ using a minimum site size of three amino acid residues, the default probe radii (1.4 and 1.8 Å), and the default connection distance (2.5 Å). Sites were manually adjusted to incorporate any residues highlighted in the literature as essential to NPS binding that were not captured by the SiteFinder tool.

#### Docking Using MOE

“Quick Prep” was used to prepare protein structures The protonation states of the NPS were established at pH 7. Docking studies were conducted with both the MMFF94 and AMBER10: EHT force fields using the GWI/WSA and dG scoring functions. A maximum of 30 poses were generated for each ligand. Early termination of the run was permitted when the all-atom RMSD between three top-ranked poses was 3.0 Å or less.

#### Docking Using GOLD

Binding sites were generated by identifying the Cartesian coordinates of the conserved aspartate residue in the MAT isoforms and selecting all residues within a 15 Å radius. GoldScore^31^ and ChemScore^32^ were used as scoring functions, and the default settings from the genetic algorithm were used to carry out the dockings.

#### Consensus Scoring

Rescoring of the docked poses using was carried out using pairwise linear potential (PLP), Poisson–Boltzmann (PB), and molecular mechanics (MM) scoring functions^33−35^ in the Galaxy/Ballaxy^36^ software. A Spearman’s rank value based on the consensus score for each of the MAT isoforms and the experimental activity of the NPS was then calculated.

#### Data Set Preparation for QSAR Models

Two data sets^5,26^ containing molecules with experimentally determined activities for the three MATs were identified. Thirty-one compounds, including NPS and other psychoactive substances, were used to construct QSAR models (see the Supporting Information).

#### Identification of Test and Training Sets

The appropriate construction and evaluation of QSAR models are reliant on training and test sets that are representative of the data set as a whole.^37^

Training sets were compiled using Tanimoto coefficients (Tc) for each MAT. A similarity coefficient matrix was produced using the open-access software OpenBabel^38^ and pairwise Tc values calculated for all molecules. The average Tc across the data set for every molecule was then obtained. Any compounds with a mean Tc of less than 0.2 were removed, as they were structurally distinct in comparison to the other molecules in the data set.

The compounds that remained were sorted according to their p*K*_i_ value into groups spanning one log unit of activity (i.e., 4–5, 5–6, 6–7, etc.). The molecules in each group with the greatest and smallest p*K*_i_ values were assigned to the training set. Of the remaining compounds in each group, the one with the greatest average Tc (i.e., the one most like the other molecules) was placed in the test set. Further assignment of molecules was carried out so that approximately 80% of the data set constituted the training set and 20% of the data constituted the test set.

Following the establishment of the test and training sets, the relative distributions of the Tc and p*K*_i_ values were interrogated to ensure that both were representative of the data set as a whole. The Shapiro-Wilks test^39^ was then used to identify whether data sets were normally distributed..

#### Descriptor Selection for QSAR Models

Using MOE, 435 physicochemical descriptors were generated for each compound. These were scaled relative to the maximum reported value in the data set. Scaled values were correlated to the compound p*K*_i_ values for each of the MATs.

Correlation coefficient (*r*^2^) with absolute values greater than 0.7 were identified, and the descriptors with the highest absolute correlation values were used to build QSAR models.

Pairs of cross-correlated descriptors with an absolute value above 0.7 were identified. The descriptor in each pair least-correlated with biological activity was removed from the study.

#### Building and Evaluating QSAR Models

QSAR models for each MAT isoform were built using QuaSAR in MOE.

The quality of each model was assessed using the correlation coefficient (*r*^2^ value) between the experimental and predicted activity and a cross-validated correlation coefficient (*q*^2^ value)

Iterative removal of the descriptors shown to contribute least to explaining the variance in experimental activity was carried out until the *r*^2^ and *q*^2^ values were similar in value and the model had the best *r*^2^ values possible with the fewest descriptors.

The models that retured the greatest *r*^2^ values with the fewest descriptors for each of the MAT isoforms (DAT, NET, and SERT) were then applied to predict the p*K*_i_ values of the molecules in the test sets using *r*^2^ as a metric to evaluate the model quality.

The extreme studentized deviate test was used on the test-set-predicted values for each model to detect outliers.

## Results

### Comparative Model Validation

The overall quality of the three comparative models, the template, and the subsequently crystal structures was analyzed using three independent but complementary protein validation tests: Ramachandran (RC) plot,^27^ Verify3D^29^ analysis, and ERRAT.^28^ These results are summarized in Table 1.

**Table 1:** Protein Validation Results for Comparative Models and Crystal Structures

structure	Ramachandran, percentage of residues in the favored or allowed region (%)	Verify3D, residues with a mean value greater than 0.2 (%)	ERRAT, quality factor (%)
4XP9 (DAT)	100	91.78	92.16
5I6X (hSERT)	100	99.53	87.67
4M48	100	94.42	93.51
DAT model Q01959	100	89.67	88.36
NET model P23975	99.7	89.24	85.77
SERT model P31645	99.8	84.17	89.45

Overall, the analysis shows that all structures are suitable for use in docking studies.

Ramachandran plots showed no violations of stereochemical quality in the MAT binding cavities, so an incorrect protein fold is unlikely to corrupt the findings of docking experiments. The small number of violations in the comparative models were restricted to loop regions This is expected, as many reported errors in comparative models result from inaccuracies in mobile loop structures.^40^ Verify3D results show structures with a high proportion of residues in favorable amino acid environments (e.g., hydrophobic residues in hydrophobic environments and hydrophilic residues in hydrophilic environments), and the ERRAT results confirm that the electronic environment of the amino acid residues, as determined by nonbonding distances between C, O, and N atoms in the structure, are generally good.

The template structure 4M48 and the experimental crystal structures (4XP9 and 5I6X) outperformed all the comparative models in each of the tests. This indicates that the models were not overfitted; hence, the consideration of how the models perform in docking studies compared to the crystal structures is meaningful.

### Docking Studies

#### Putative Binding Site Identification

**Figure 1:**
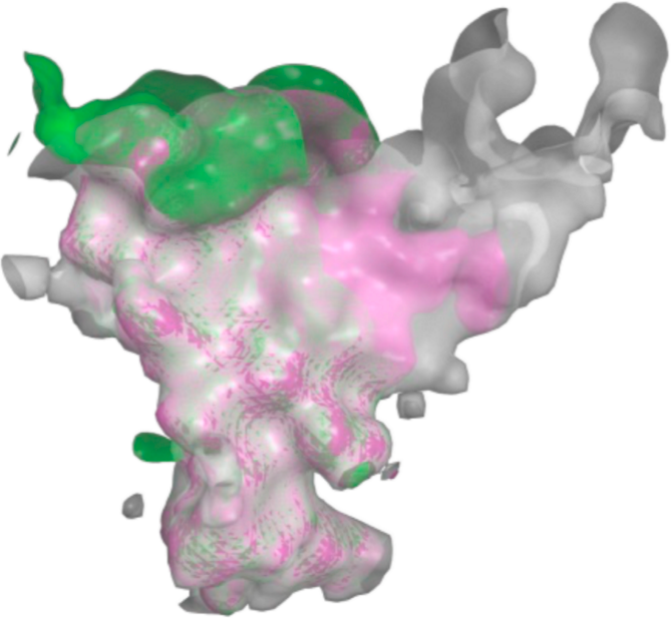
Overlaid putative binding sites of the DAT (green), NET (white), and SERT (magenta) MAT homology models illustrating the similarities in the size and shape of the putative binding cavities.

Putative binding cavities were identified for the MAT comparative models (Figure 1). To ensure the incorporation of residues known to be implicated in biological response, the composition of each site was conducted by cross-referencing with the literature.^7,41,42^ These sites were shown to be druggable via their respective propensity for ligand binding (PLB) scores and volumes (DAT, 3.8 and 270 Å^3^; NET, 4.25 and 341 Å^3^; and SERT, 3.5 and 249 Å^3^)

#### Docking of Native Substrates

Norepinephrine, serotonin, and dopamine were docked into the comparative MAT models (Table 2).

**Table 2:** Baseline S-Score Values for the Docking of Native Substrates into the MAT Comparative Models

	dopamine	norepinephrine	serotonin
DAT (Q01959)	–4.6682	–4.7047	–4.7057
NET (P23975)	–4.7191	–4.9835	–5.4317
SERT (P31645)	–5.0198	–4.9049	–5.3776

These docking studies identify a number of amino acids previously established as key with regard to the formation of protein–ligand interactions in DAT.^20,43^ A number of drugs, e.g., citalopram have been shown to form hydrogen bonds with Asp79 and Asp 476 in DAT.^44,45^ These interactions were replicated in the docking studies with dopamine.

The docking of norepinephrine in the NET model reproduces the experimental observations of Schlesinger and co-workers,^21^ who highlighted Asp75, Phe72, Tyr152, and Phe317 as being important to protein–ligand binding.

The docking of serotonin in the SERT model also shows reproduces experimental observations, including key interactions with Asp 98 and Tyr 95.^46^ The X-ray crystal structures of human SERT bound to paroxetine (45) and several cocrystallized dDAT structures^45^ are available in the public domain. The human SERT structure (PDB accession code 5I6X) showed a binding pocket containing Ile172, Tyr176, Phe335, and Ser438 along with potential hydrogen-bonding interactions between paroxetine and both Tyr95 and Asp98, as predicted by the docking studies with the SERT model.

These findings together offer reassurance that the models are appropriate for use in docking studies. However, it should be noted that the selectivity of MATs for their preferred transporter was not clear from the *S* values returned. Even at this early stage, this speaks to the challenges associated with the promiscuity of the MATs and production of a model that can distinguish between them.

#### Docking of the Iversen Data set

Thirty-one NPS described by Iversen and co-workers were docked into the MAT models using two independent docking algorithms, namely, MOE and GOLD.^48^

The dDAT crystal structure complexed with d-amphetamine (PDB accession code 4XP9),^45^ was selected from the 4X series to use in the docking studies as it had the highest resolution (2.80 Å). Similarly, the human SERT crystal structure complexed with *s*-citalopram (5I6X) was used as it had the highest resolution (3.14 Å).^45^

Results (Table 3) showed no direct correlation between the *S* value, GoldScore, ChemScore, and the p*K*_i_ (*r*^2^ ranging from 0.000 to 0.152). This observation is not novel. It is well-documented that protein–ligand interactions are complex; thus, the ability of a single scoring function to correctly account for the inherent complexity in protein–ligand interaction that gives rise to the experimental p*K*_i_ is necessarily limited.^49^ As such, Spearman’s rank was used to determine if there were correlations between the relative ranking of the experimental activities and the scoring function values.

**Table 3:** Correlation and Spearman Ranking Analysis Results for Comparative Models of DAT, NET, and SERT and X-ray Crystal Structures of DAT and SERT

	docking algorithm, forcefield or scoring function	DAT (Q01959)	NET (P23975)	SERT (P31645)	SERT (5I6X)	DAT (4XP9)
correlation coefficients (*r*^2^)	MOE, AMBER10	0.146	0.017	0.082		
	MOE, MMFF94x	0.006	0.078	0.033		
	GOLD, GoldScore	0.021	0.017	0.152		
	GOLD, ChemScore	0.017	0.000	0.000		
	MOE, London Δ*G*	0.38	0.13	0.30		
Spearman ranking (ρ)	MOE, affinity Δ*G*	0.42^a^	0.30	0.28	0.48^b^	0.42^a^
	MOE, ASE	0.34	0.05	0.13		
	MOE, αHB	0.40^a^	0.08	0.28		
	MOE, E_place	0.22	0.03	–0.03		
	MOE, E_conf	0.25	–0.02	–0.12		
	GOLD, GoldScore	0.40^a^	0.04	0.19		
	GOLD, ChemScore	0.28	0.19	0.24		
	Ballaxy, MM	–0.22	0.04	0.23		
	Ballaxy, PB	0.27	0.12	0.11		
	Ballaxy, PLP	0.41^a^	0.07	0.23		
	consensus score	0.38^a^	0.05	0.40^a^		

a Statistically significant Spearman ranking results at 90% confidence.

b Statistically Significant Spearman ranking results at 95% confidence.

Consensus scoring, an established technique for improving the degree of correlation between scoring function predictions and experimental values,^50,51^ was carried out. It is predicated on reducing the bias in any individual scoring function by combining and contrasting the results obtained from complementary but independent algorithms.

Scoring functions from MOE (London Δ*G*, affinity Δ*G*, ASE, and αHB), GOLD (GoldScore and ChemScore), and Ballaxy^35^ (MM, PLP, and PB) were used to identify the average consensus rankings for each of the 21 NPS in the docking experiments.

Consensus rankings were used as inputs in a Spearman ranking analysis. In such an analysis, a statistically significant result is demonstrated when a threshold value (determined by the size of the data set) is exceeded. The values reported do not correspond to correlation coefficients. This approach showed statistically significant correlations at 90% confidence between ranked experimental activity and consensus rankings for DAT and SERT. No statistically significant correlation was observed for NET (Table 3)

Docking studies with the experimental structures showed improvement over the comparative models in terms of the Spearman’s correlation coefficient for DAT (from 0.38 to 0.42), but both experiments showed identical levels of confidence in the rankings (90%). The Spearman’s correlation coefficient for SERT also increased (from 0.30 to 0.48), increasing the confidence level from 90% to 95% as a result (Table 3).

#### Identification of a Diverse Training Set for QSAR Studies

The distribution of molecules in the QSAR training sets according to two different diversity metrics, namely, FPMACCS and Tanimoto, was investigated using the experimentally derived p*K*_i_ for DAT as the discriminant (Figure S1)

The Tanimoto-derived training set is a more complete representation of the data set, as the distribution of molecules across the one log unit activity range is proportionate to the distribution across the data set unlike the FPMACCS-derived training set, which attributes a disproportionate number of compounds in the p*K*_i_ 5–6 range to the training sets. Therefore, test and training sets for all models were established using the Tanimoto method.

The diverse training and test sets for the MATs identified using Tanimoto coefficients in conjunction with experimental activity are summarized in Table 4. p*K*_i_ values vary for each compound and MAT isoform. Therefore, the training sets and test sets for DAT, NET, and SERT are different.

**Table 4:** Psychoactive Substances That Constitute the Test and Training Sets Used in the QSAR Studies of the MAT Isoforms

DAT		NET		SERT	
training set	test set	training set	test set	training set	test set
5-APB, 5-iodo-aminoindane, benzedrone, bupropion, citalopram, cocaine, desipramine, desoxypipradrol, fluoxetine, GBR 12935, imipramine, MDAT, methiopropamine, methylethcathinone, MNB-cathinone, naphyrone, nisoxetine, nomifensine, amitriptyline, *R*-MDMA, RTI-55, *S*-amphetamine, *S*-MDMA, WIN 35428	6-APB, nortriptyline, mazindol, MDAI, mephedrone, 1-naphyrone	1-naphyrone, 5-APB, 6-APB, amitriptyline, benzedrone, cocaine, desipramine, desoxypipradrol, GBR 12935, imipramine, MDAD, MDAT, mephedrone, methiopropamine, methylethcathinone, MNB-cathinone, naphyrone, nisoxetine, nomifensine, nortriptyline, *R*-MDMA, RTI-55, *S*-amphetamine, *S*-MDMA	5-IAI, bupropion, citalopram, fluoxetine, mazindol, WIN 35428	1-naphyrone, 5-APB, 5-iodo-aminoindane, 6-APB, benzedrone, citalopram, desipramine, desoxypipradrol, fluoxetine, GBR 12935, mazindol, MDAI, MDAT, mephedrone, methiopropamine, methylethcathinone, MNB-cathinone, nisoxetine, nortriptyline, RTI-55, *R*-MDMA, *S*-amphetamine, *S*-MDMA, WIN 35428	amitriptyline, bupropion, cocaine, imipramine, naphyrone, nomifensine

#### Data Set Preparation

Average pairwise Tanimoto coefficients (Figure S2) were calculated to identify any molecules in the data set that were significantly different in terms of chemical structure compared to the data set. This is important because it is impossible for QSAR models to meaningfully predict the activities of structurally distinct molecules. the incorporation of such compounds into the test or training sets could give rise to insights into the predictivity and robustness of the QSAR. As such, any molecule with an average Tc value less than 0.2 was removed from the data set.

One compound, dimethylamylamine (Table S1, compound 9) had a value below this threshold 0.17 (±0.15). It was removed before the QSAR models were built and tested.

### Building and Evaluating QSAR Models

#### DAT QSAR model

The incremental iterative approach to descriptor selection resulted in a model built from three variables (eq 1) that generated *r*^2^ = 0.68 and *q*^2^ = 0.51. This suggests that the model, because of the small number of descriptors used, should be generalizable and that it is predictive and robust for the training set.

Prediction of experimental activities in the test set returned *r*^2^ = 0.63. This further demonstrates that the model is predictive but not overfitted.
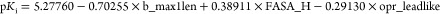
1Here b_max1len is the length of the single-bond chain in the molecule, FASA_H represents the water-accessible surface area of the molecule, and opr_leadlike is a binary value that demonstrates whether two or fewer (1) or three or more (0) of the lead-like criteria in a molecule are violated.

#### NET QSAR Model

Again, a three-descriptor model (eq 2) was found to be the best-performing model for the training set (*r*^2^ = 0.6). However, the robustness of the model was significantly poorer when compared to that of the DAT model (*q*^2^ = 0.39), and the predictivity of the model with respect to the test set was also very poor (*r*^2^ = 0.1). This indicates that the generation of a robust and predictive NET QSAR model was not achieved.
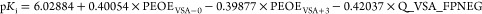
2Here PEOE_VSA-0_ is the van der Waals surface area on the molecule with partial charges between −0.05 and 0, PEOE_VSA+3_ is the van der Waal surface area on the molecule with partial charges between 0.15 and 0.20, and Q_VSA_FPNEG is the fractional negative polar van der Waals surface area of the molecule.

#### SERT QSAR Models

The best-performing SERT QSAR model was a robust three-descriptor model (eq 3, *r*^2^ = 0.87 and *q*^2^ = 0.80). This model was also shown to be predictive (test set *r*^2^ = 0.74) without being overfitted.
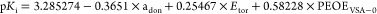
3Here a_don is the number of hydrogen-bond-acceptor atoms in the molecule, *E*_tor_ describes the torsional potential energy of the molecule, and PEOE_VSA-0_ is the van der Waals surface area on the molecule with partial charges between −0.05 and 0

## Discussion

### Comparative Model and X-ray Crystal Structures Docking Studies

The availability of comparative models for all three MAT isoforms, experimental structures for the dDAT crystal structure complexed with d-amphetamine (PDB accession code 4XP9, resolution of 2.8 Å),^45^ and an X-ray structure for the human serotonin transporter complexed with the antidepressant *s*-citalopram (PDB accession code 5I6X, resolution of 3.15 Å).^47^ afforded the opportunity to compare results from the docking experiments with DAT (Q01959) and SERT (P31645) to those with experimental crystal structures and examine the limitations of comparative models under these circumstances.

The 4M48^45^ template used to construct the DAT (Q01959) and SERT (P31645) comparative models has amino acid sequence identities with the MAT isoforms of between 53 and 55%. It is widely accepted that a model with at least 50% sequence identity to its template can be used meaningfully to investigate potential protein–ligand interactions.^52^

**Figure 2:**
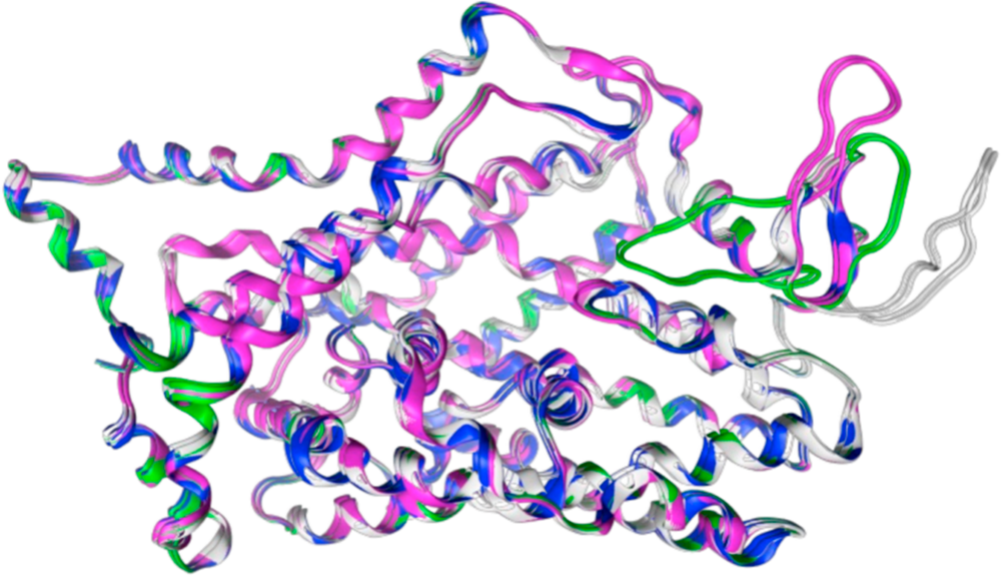
Superimposed Ca traces of 4M48 (blue), Q01959 (DAT, green), P23975 (NET, white), and P31645 (SERT, magenta) illustrating a conserved tertiary structure with variations in loop regions.

When comparing the structures, the overall protein fold and secondary structures are highly conserved (Figure 2). This indicates that any selectively that does arise is unlikely to be due to differences in gross structural differences, which may pose challenges when attempting to rationalize the selectivity between the different MATs from the docking studies.

Molecules from the Iversen data set were observed to bind toward the bottom of the cavities in each of the MAT isoform models. This is likely driven by the interaction of a conserved aspartate residue (Asp79 in Q01959, Asp75 in P23975, and Asp98 in P31645) with an amino functionality on the ligands. This conserved aspartate residue has been shown to important in key interactions between the biogenic amines dopamine, norepinephrine, and serotonin^4,17,21,41,45,53−57^ in addition to a large number of known inhibitors of the MAT isoforms.

As a result of the acknowledged lack of correlation between experimental activity and scoring function values, several docking studies have used Spearman’s rank to interrogate the relative rankings of docking poses.^58^ For this set of experiments (21 compounds), ρ values above 0.37 and 0.44 are considered significant at 90% and 95% confidence, respectively.^59^

Statistical analysis showed no significant correlation for any of the MATs between the experimental values and any of the scoring functions at 95% confidence (Table 3). However, there was significant correlation between experimental activities and the London Δ*G*, affinity Δ*G*, αHB, and GoldScore functions at 90% confidence for dockings in the DAT (Q01959) model.

The obtained results illustrate the inherent challenge identified because of the similarities between the MATs. To gain reassurance that the results did not arise because of biases or limitations in individual scoring functions, high-ranking poses from the docking experiments were rescored using Ballaxy^34^ using the MM, PB, and PLP scoring functions.^33,34,60^ A Spearman’s ranking analysis incorporating each of the Ballaxy functions was then conducted.

The results from the rescoring experiment show a significant relationship at 90% confidence between PLP and experimental activities for the docking of the data set into the DAT model but do not improve on the previous results. This suggests that it is unlikely that a single scoring function is going to be able to provide insight into selectivity between DAT, NET, and SERT. Consensus scoring, which looks at average relative rankings across the scoring functions, did yield statistically significant results for the DAT and SERT dockings at 90% confidence. This speaks to the limitations of individual scoring functions in discrimination at a fine-grained level and the benefits of consensus scoring in ameliorating bias, which are supported by previous studies that have shown that using consensus scoring methodologies improves the correlation for ranked data.^61^

Why the consensus approach did not show an improvement for NET similar to that observed for SERT is unclear. It is possible that the use of comparative models, as opposed to experimental structures, could have negatively impacted the ability of the consensus score methodology to predict the NET ranked data, but if this was the case similar failings could have been expected for DAT and SERT given the homologous nature of the MAT isoforms. Another potential cause could be the relatively small data set used in the docking study. Many consensus score studies use between 100^54,56^ and 1000 ligands.^51^ This means that the relatively limited variation in experimentally observed binding values for the 21 ligands in the Iversen data set may render relative rankings arbitrary and could explain why docking results are not discriminative. However, if this were the explanation, it might again be expected that similar results would be observed for the DAT and SERT docking studies.

Putting these arguments to one side, given the overall similarity in shape and size of putative binding cavities, any differences observed between the MAT models must arise due to variations in the cavities at the residue level.

Examination of the amino acid sequence alignments of DAT, NET, and SERT (Figure S3) shows that there is a high level of conservation across the isoforms in the amino acids near the conserved aspartate residue. Therefore, the binding site compositions between the isoforms are expected to be similar. Analysis of binding site residues at the 1D level shows a high degree of similarity between the isoforms, e.g., nonidentical but hydrophobic residues. In addition, no differences in amino acid conformation were observed at the 3D level with respect to orientation of conserved residues in the MAT homology models, most likely because they were derived from the same template.

This similarity between the binding cavities of the MAT isoforms at the 1D and 3D levels contextualises the difficulties encountered in developing discriminative docking models. It also is evidence to suggest that while comparative models are invaluable for providing testable hypotheses, there may be limitations when attempting to understanding selectively between isoforms, particularly where the isoform models are generated from the same initial templates.

### Comparison to X-ray Crystal Structures

The crystal structure of the human serotonin transport structure 5I6X(45) was validated (Table 1) and used in docking studies. This did not result in a significant increase in correlation between predicted and experimental activities when compared to the results from the SERT (P31645) study. Comparison of the crystal structure and the model shows a protein backbone RMSD value of 2.65 Å. However, the major variations between the two structures are evident in extracellular loop regions, with the relative positions of secondary structure elements remaining largely conserved. This helps aid the post hoc rationalization of the lack of difference in the results of the studies.

However, closer examination of the dockings showed there was a significant difference in the lowest-energy conformation for fluoxetine docked into SERT (P31645) compared to the X-ray crystal structure. The X-ray crystal structure was seemingly able to accommodate fluoxetine deeper in the binding cavity than the comparative model.

Interrogation of the structures shows 5I6X has a narrower entrance to the innermost section of the binding site (approximately 6 Å in diameter) when compared with P31645 (9.6 Å at the narrowest point and 13.6 Å at the widest point).

**Figure 3:**
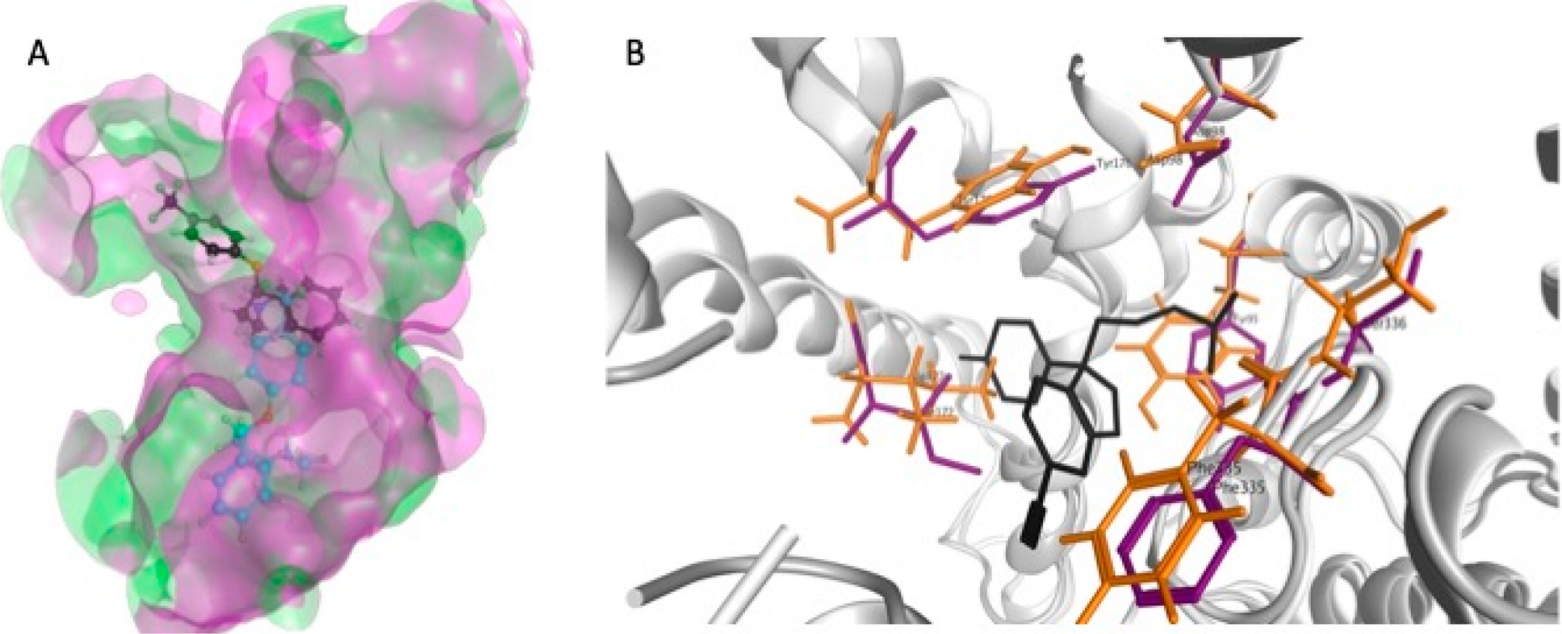
(A) Overlaid binding sites of P31645 (green) and 5I6X (magenta) with docked fluoxetine (black ball and stick and cyan ball and stick, respectively) showing differences in ligand position. (B) Overlaid binding sites of P31645 (orange) and 5I6X (purple) illustrating the variation in side-chain conformation. Co-complexed *s*-citalopram (black stick model) from 5I6X is shown for reference.

The conformations of the binding site residues in P31645 and the 5I6X also differ. This gives rise to this subtle but significant alteration in the topology of the binding site (Figure 3).

Spearman rank analysis of the docking studies performed using the SERT homology model generated a ρ value of 0.30 that was not significant at 90% confidence. However, the studies with 5I6X generated a ρ value of 0.48, which was significant at 95% confidence. This may be because the narrowing of the cavity at the deepest part of the binding cavity in the crystal structure provides an inherent steric constraint for the placement algorithm that prevents the docking algorithms from returning favorable scores for molecules positioned toward the top of the cavity. Hence, differences are easier to detect because the positions of the docked compounds in the 5I6X cavity are less variable, meaning that changes in scores are more attributable to the differences in the structure of the ligands than the studies carried out in the SERT homology model, which has a more open cavity and hence fewer inherent constraints.

dDAT crystal structures are also available in the public domain. Docking studies were conducted using the 4XP9 crystal structure (2.8 Å resolution) cocrystallized with d-amphetamine^45^ to determine whether similar gains in ρ values for DAT could be obtained when the crystal structure was used rather than the homology model. Although ρ values did improve (from 0.38 to 0.42), there was not a significant difference. This was rationalized post hoc by the fact that superimposition of 4XP9 and the DAT (Q01959) comparative model showed that the backbones were almost identical (RMSD 0.729 Å) and that the binding cavities were very similar.

No equivalent NET crystal was available in the public domain at the time of writing, so no comparable experiment was possible for this MAT isoform.

### Analysis of QSAR Models for the MAT Isoforms

Relative distributions of experimental p*K*_i_ values for the compounds used in this study with reference to the MAT were analyzed (Figure S4). SERT has the greatest range of activities (seven log units). The spans of activity values for DAT and NET are smaller (five log units for both).

Experimental p*K*_i_ values are normally distributed for DAT and SERT at 99% confidence. Experimental values for NET are not normally distributed even at a 90% confidence limit (i.e., the data are skewed). This may negatively influence the predictivity of any models generated for NET.

For the DAT QSAR model, activity values in the training and test sets were normally distributed as per the Shapiro–Wilks Test (*p* = 0.902, *W* = 0.967, H0 is accepted) and the relative distribution of p*K*_i_ values in the test and training sets was reflective of the distribution of experimental activity in the data set with one exception. Only one compound in the DAT data set had an experimental activity greater than p*K*_i_ 8. This means that the test set did not contain any compounds with a p*K*_i_ between 8 and 9, whereas the training set did.

Activity values for NET ranged between p*K*_i_ 4 and 9. However, these values were not normally distributed even at 90% confidence (*p* = 0.902, *W* = 0.876, H0 was rejected). Twenty-three of the compounds have a p*K*_i_ value between 5 and 7. One compound has a p*K*_i_ between 4 and 5, three compounds have p*K*_i_ values between 7 and 8, and four compounds have activity values between 8 and 9. This skew was accounted for in the generation of the test and training sets for the NET QSAR. Both training and test sets that mirrored the p*K*_i_ distribution across the data set were identified. It was postulated that an approach of this nature would provide the best opportunity to generate a QSAR model for NET that was both robust and predictive despite the inherent limitations of such a skewed data set.

The activity data for SERT were normally distributed (*p* = 0.902, *W* = 0.957, H0 is accepted), spanning from p*K*_i_ 3 to 10. The training and test sets generated to construct the QSAR models were shown to represent the range and distribution of experimental activities across the whole data set.

Current best practice suggests the maximum number of descriptors for a QSAR model should not be more than one descriptor per five compounds This means that the models constructed for these experiments should comprise no more than four descriptors.

Relative distributions of experimental activity values appear to impact on the ability to generate robust and predictive QSAR models for the MAT isoforms. Where normal distributions in the overall data set and training and test sets were observed (DAT and SERT), it was possible to generate models that were predictive and robust. Where this was not the case (NET), the predictive ability of the QSAR models was significantly curtailed and the models were not robust.

### Post Hoc Analysis of QSAR Models

#### DAT

Considering the DAT QSAR model provides the following insights. Given that the descriptor values are scaled, the negative coefficients that preceed the b_max1len and opr_leadlike descriptors indicated that these were penalty terms.

b_max1len captures the length of the longest single-bond chain in a molecule as an integer value. Given that this is a penalty term, longer chain lengths will generate smaller predicted p*K*_i_ values, while shorter chain lengths will result in greater predicted activity values. This could be explained if entropic arguments are considered. Molecules with shorter single-bond chain lengths are likely to be more rigid than those with longer single-bond chain lengths. The shorter-chain-length molecules will therefore have a smaller entropic penalty to receptor binding and are predicted by the model to bind more tightly than larger, more flexible molecules. This term can also be related back to the three-dimensional structure of the receptor. Analysis of the Q01959 binding cavity^62^ shows a narrowing in the cavity from 10 Å at the mouth to 7 Å at the deepest part of the pocket where the compounds bind, as shown by empirical evidence and docking studies. Smaller, less flexible molecules may be better able to access and interact with the deepest parts of the cavity, which explains why the b_max1len descriptor is incorporated into the DAT QSAR model.

The second descriptor, FASA_H, represents the water-accessible surface area of the hydrophobic atoms in a molecule. The positive coefficient preceding FASA_H in the model implies that a larger hydrophobic surface area will result in higher predicted p*K*_i_ values.

Several of the amino acid residues comprising the binding site in DAT are hydrophobic (e.g., L80, A81, V152, F320, and F326). It follows that interactions with the hydrophobic surface area of the with hydrophobic atoms in the ligand could be favorable to protein ligand binding, which gives context as to why FASA_H is an important factor to explain the difference in binding affinities for small molecules with DAT.

Energetic arguments would also support the displacement of labile water molecules from the hydrophobic binding cavity and their replacement with hydrophobic protein–ligand contacts, i.e., the greater the water accessible surface area and hence potential hydrophobic contacts, the higher the p*K*_i_. This means that a hydrophobic compound that can form multiple contact with the deepest part of the DAT binding cavity, which is also predominantly hydrophobic, will be predicted to have a higher p*K*_i_ than one that only partially fills it, providing context as to why FASA_H was highlighted as being important in the DAT QSAR model

A molecule can have one of two values for the final variable in the DAT QSAR model, i.e., opr_leadlike descriptor: 1, which indicates fewer than or equal to 2 violations of the Oprea^63^ lead-like criteria, and 0, which indicates compounds with three or more violations. This term is a penalty term in the DAT model, suggesting that molecules that violate three or more of the Oprea lead-like criteria are preferred, which counterintuitively implies that molecules that are not drug-like will preferentially bind DAT compared to those that are.

All the compounds in the training set, except for GBR 12935, had an opr_leadlike value of 1. The descriptor is a composite function, and opr_leadlike values provide no insight into which of the composite terms were violated. Arguably, this means that opr_leadlike is most likely a “correction factor” in the DAT QSAR model. This hypothesis was tested by deleting the descriptor from the model and regenerating a 2two-descriptor model using only b_max1len and FASA_H. Results from this model show values of *r*^2^ = 0.67 and *q*^2^ = 0.50 for the training set, which are comparable to those of the three-descriptor model; however, the *r*^2^ value for the test set decreased from 0.63 to 0.35.

The fact that the three-descriptor model outperforms the two-descriptor model is evidence that opr_leadlike is important for predictivity as a correction factor. To further probe the corrective nature of opr_leadlike, a modified two-descriptor equation was applied (eq 4) to the DAT data set. If opr_leadlike was genuinely a correction factor, it could be substituted by a constant value (−0.29310) with little impact on the predictivity of the model.
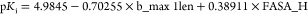
4

Application of eq 4 to the data set leads to predicted p*K*_i_ values that are identical to those from the three-descriptor model for compounds where opr_leadlike is 1 and overprediction for the handful of molecules where the opr_leadlike value is 0, lending further credence to the fact that the descriptor indeed functions as a correction factor and does not have further meaning in terms of understanding binding to DAT.

#### NET

Descriptor selection only returned an electronic descriptor for the NET QSAR model. The best-performing three -descriptor model that was generated was neither predictive (test set *r*^2^ = 0.1) nor robust (*r*^2^ = 0.6, and *q*^2^ = 0.39) even though time was taken to ensure that both training and test sets mirrored the data set as a whole and none of variables used in the construction of the QSAR were cross-correlated. Therefore, it is unlikely that the model underperforms because of a lack of due diligence during its construction.

The composition of the data set could explain the underperformance of the QSAR models. In contrast to the SERT and DAT data sets, the experimental activity values for NET span a smaller range. Analysis showed that the data for NET were not normally distributed. Most of the compounds (23 of 31) have p*K*_i_ values between 5 and 7. This clustering of compounds impacts on how the training set and test sets are constructed. It becomes inherently more challenging to identify physicochemical properties responsible for variations in activity, which subsequently impacts how predictive any QSAR model can be. This limitation was noted when the experiment began, and mitigations were put in place to ensure the identification of test and training sets that were representative of the data set. Despite these measures, it was not possible to generate a predictive and robust QSAR model for NET. Therefore, it must be concluded that the small data set and the narrow range of p*K*_i_ values limit the ability to generate a robust and predictive model. This is a known problem in the construction of QSAR models.^64^

#### SERT

Of the three MATs, SERT generated the most predictive QSAR models (*r*^2^ = 0.87, and *q*^2^ = 0.80). The activity values in SERT were spread across a wide range, which is ideal for producing generalizable QSAR models.^25^

PEOE_VSA–0_ was shown to be the most important descriptor in the SERT model. Considering the positive descriptor coefficient for PEOE_VSA–0_, this implies that higher affinity will exist in compounds with neutral or weakly negative values. This suggests that hydrophobic interactions between the ligand and the receptor will be important in determining the degree of affinity with SERT. In this respect, the finding is similar to that for the DAT model, which placed an emphasis on hydrophobic interactions to determine receptor–ligand binding.

a_don describes the number of hydrogen-bond-donor atoms in a compound, excluding atoms that are basic but including moieties that can function as either an acceptor or a donor. a_don is a penalty term in the SERT QSAR model by virtue of the negative coefficient. This means that activity will be higher when molecules have fewer hydrogen bond donors. As such, beyond the biogenic amine groups, which are a common feature of the molecules in the data set, there should ideally be no further hydrogen-bond donors in the molecule. This aligns with the relative importance of PEOE_VSA–0_ in predicting biological activity in SERT, further supporting that hydrophobic molecule will bind most strongly.

The final descriptor in the equation is *E*_tor_ which describes the torsional potential energy of a molecule. All *E*_tor_ values are positive when calculated. Flexible molecules have smaller values, and more rigid molecules have larger values. Analysis of the QSAR implies that the binding affinity to SERT will be greater for rigid molecules than more flexible ones.

This observation is of considerable interest, as it demonstrates striking similarities between the findings for the DAT and SERT models. Although the explicit identities of the descriptors are different between the two models, in both cases the descriptors are proxies for two overarching features. That is, for binding to occur to these MATs, molecules need to be hydrophobic (except for the biogenic amine group) and rigid. These similarities provide insight into the promiscuity of ligand binding between the MATs and re-emphasizes the significant challenge in identifying factors to account for the differences in binding affinities between the isoforms because of these similarities.

## Conclusions

This study aimed to determine whether in silico structure-based and ligand-based methodologies could provide insight into selectivity for the monoamine transporters DAT, NET, and SERT. Such insight could facilitate the development of MAT-selective therapeutics such as SSRIs or give insight into potentially novel chemical scaffolds that, although currently unexploited, could emerge in the future as NPS.

The overall amino acid sequence between the MATs was more than 50% identical, which increased to 75% when comparing the binding sites. This gave early indications that the structures were highly similar and hence that rationalizing selectivity at the molecular level could be challenging.

Although a series of independent yet complementary validation steps showed that the MAT structures were sufficient for use in docking studies, novel insights that arose as a consequence of this study include the fact that docking experiments did not provide insight into the molecular basis for the difference in activities for the NET comparative model.

The docking experiments carried out on the DAT and SERT homology models did show correlation between experimental activity and consensus scores. However, these results did not provide further insight into how the differences in affinity between these isoforms for the same molecules had arisen.

Subsequent experiments carried out on the X-ray crystal structures for DAT and SERT illustrated some of the limitations of comparative models in docking studies by demonstrating how small differences can impact on the architecture of putative binding cavities and influence the results of docking experiments.

Previously undescribed QSAR models that were both robust and predictive were constructed for DAT and SERT. The SERT model performed best most, likely because of the diverse range of experimental p*K*_i_ values associated with the isoform (range of 7 log units). As a direct consequence of these studies, it was discovered that it was not possible to identify a robust and predictive QSAR model for NET, which was likely a consequence of the skew of the underlying data set, i.e., the over-representation of compounds with p*K*_i_ values between 5 and 7.

From both the docking and QSAR studies, it is evident that can be there are structural similarities in the binding cavities that may explain both the degree of promiscuity between the monoamine transporters for the data set investigated and the similar physicochemical properties shown as important for binding to DAT and SERT from the QSAR descriptors. The novel DAT and SERT QSAR models suggest that compounds should be relatively inflexible and have hydrophobic surface areas to optimize interaction between the ligand and the binding sites. These key findings should be considered to contextualize the considerable challenges in developing both compounds that are selective for one MAT over another and the computational models that are able to rationalize these differences.

## Notes

Molecular Operating Environment is available via the Chemical Computing Group for Linux, macOS, and Windows for licensed users and free evaluation purposes at https://www.chemcomp.com/Contact.htm?q=trial_request. GOLD is available via the Cambridge Crystallographic Data Centre for licensed users and evaluation purposes at https://www.ccdc.cam.ac.uk/solutions/for-academia/. Ballaxy is available for download at https://ball-project.org/.

## Supporting Information Available

Names, experimentally determined p*K*_i_ values, chemical structures, and classifications of the psychoactive compounds used in the studies (PDF)
