# Supplementary material for: In Silico Investigations into the Selectivity of Psychoactive and New Psychoactive Substances in Monoamine Transporters
Source: ACS Omega. 2022 Oct 21;7(43):38311–21. doi: 10.1021/acsomega.2c02714 (PMC9631908; doi:10.1021/acsomega.2c02714)
Supplement: Supplementary file 1 — ao2c02714_si_001.pdf [file ao2c02714_si_001.pdf]

In silico investigations into the selectivity of psychoactive and new psychoactive substances (NPS) in the monoamine transporters

*Michelle J. Botha and Stewart B. Kirton**

Department of Clinical Pharmaceutical and Biological Science, University of Hertfordshire, Hatfield, Hertfordshire, United Kingdom AL10 9AB.

Table S1: Compounds used in this study, their biological activity against the MATs, chemical structure and NPS classification.

Compound	pKi DAT	pKi NET	pKi SERT	Structure	Classification
1. Mephedrone	6.08	5.88	4.99	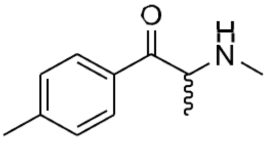	Cathinone
2. Methiopropamine	6.05	6.09	4.14	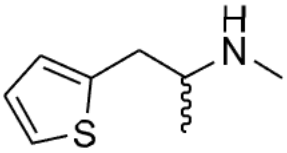	Phenethylamine
3. Methylenedioxy-N-benzylcathinone (MNB-cathinone)	6.01	5.37	4.56	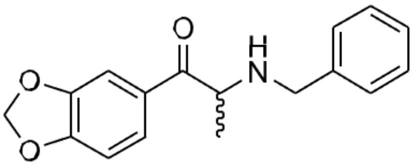	Cathinone
4. 5-APB (1-(benzofuran-5-yl) propan-2-amine)	6.30	6.33	5.78	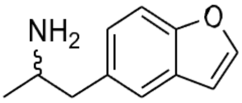	Phenethylamine
5. 6-APB (1-(benzofuran-6-yl) propan-2-amine)	6.63	6.52	5.26	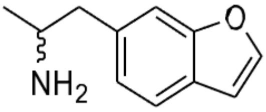	Phenethylamine

6. Desoxypipradrol	7.30	6.26	4.27	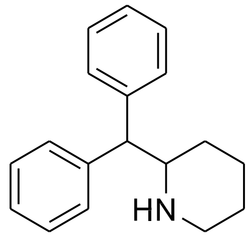	Piperidines & Pyrrolidines
7. 5-Iodo-2-aminoindane (5-IAI)	5.61	6.09	5.75	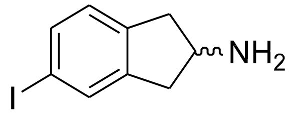	Aminoindane
8. Benzedrone	5.64	5.50	4.75	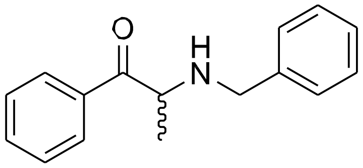	Cathinone
9. Dimethylamylamine	4.74	5.77	3.75	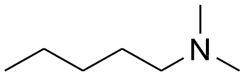	Others
10. Methylenedioxy-Aminoindane (MDAI)	5.12	5.78	4.93	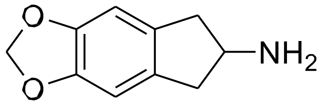	Aminoindane
11. Methylenedioxy-Aminotetralin (MDAT)	5.20	5.64	5.65	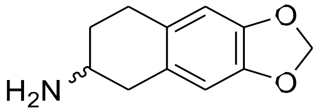	Phenethylamine
12. Naphyrone	7.28	6.70	6.63	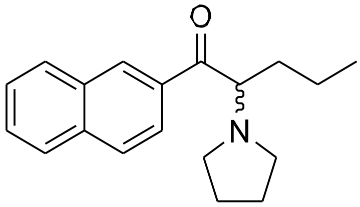	Cathinone
13. 1-Naphyrone	7.32	6.27	6.45	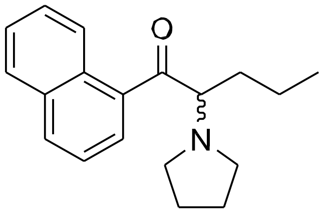	Cathinone
14. Methylethcathinone	6.08	5.36	5.43	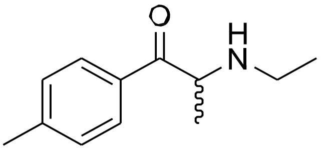	Cathinone

15. Amitriptyline	4.61	7.00	7.83	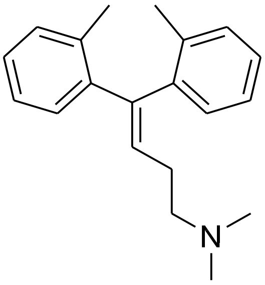	Others
16. Nomifensine	6.90	7.07	5.56	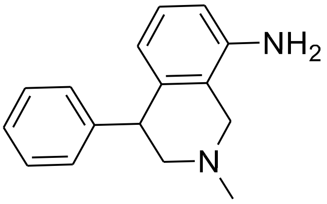	Others
17. Cocaine	6.19	5.20	5.62	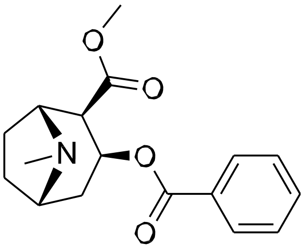	Others
18. R-MDMA	5.21	5.50	5.17	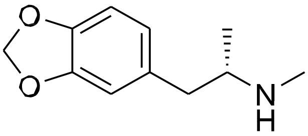	Phenethylamine
19. S-MDMA	5.62	5.70	5.56	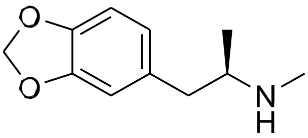	Phenethylamine
20. S-Amphetamine	6.54	6.20	4.78	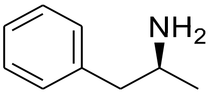	Phenethylamine
21. Fluoxetine	5.04	5.20	6.55	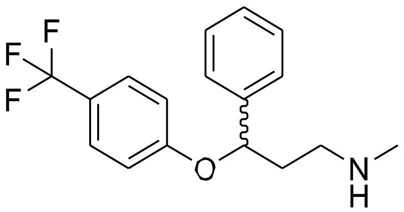	Others

22. RTI-55	8.49	8.60	9.31	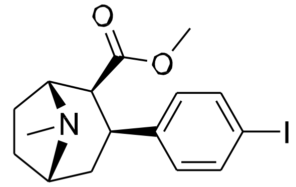	Others
23. WIN 35428	7.58	7.50	6.90	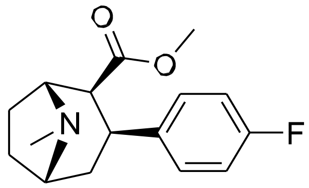	Others
24. GBR 12935	7.67	6.65	5.19	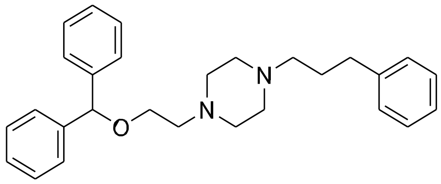	Piperazine
25. Bupropion	5.56	5.86	4.35	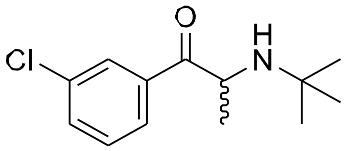	Cathinone
26. Nisoxetine	6.32	8.29	6.42	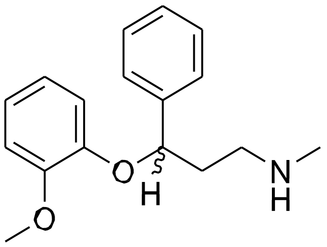	Others
27. Desipramine	4.10	8.40	7.21	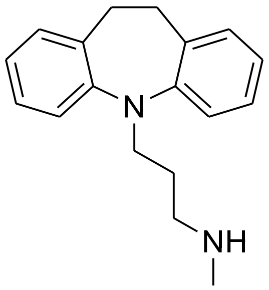	Others

28. Nortriptyline	4.86	8.47	6.79	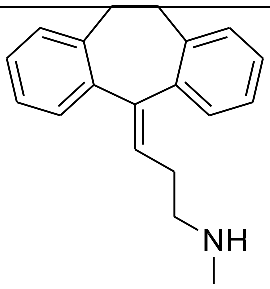	Others
29. Mazindol	7.56	8.49	6.82	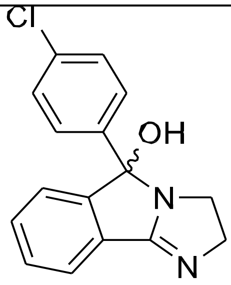	Others
Compound	DAT	pK_i<br>NET	SERT	Structure	Classification
30. Imipramine	5.01	7.17	8.11	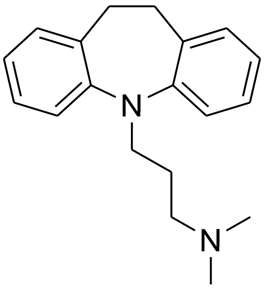	Others
31. Citalopram	5.00	6.00	8.27	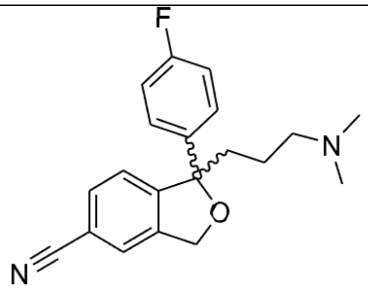	Others

Table S2: List of New Psychoactive Substances used in the study with SMILES strings.

NPS name	SMILES
Desoxypipradrol	C(c1ccccc1)(c1ccccc1)C1NCCCC1
Buproprion	Clc1cc(C(=O)C(NC(C)(C)C)C)ccc1
Mazindol	Clc1ccc(C2(O)N3C(=NCC3)c3c2cccc3)cc1
Fluoxteine	FC(F)(F)c1ccc(OC(CCNC)c2ccccc2)cc1
Citalopram	Fc1ccc(C2(CCCN(C)C)OCc3c2ccc(C#N)c3)cc1
WIN 35,428	Fc1ccc([C@@H]2[C@H](C(=O)OC)[C@@H]3N(C)[C@H](C2)CC3)cc1
5- Iodoaminoindane (5-IAI)	Ic1cc2c(cc1)CC(N)C2
RTI-55	Ic1ccc([C@@H]2[C@H](C(=O)OC)[C@@H]3N(C)[C@H](C2)CC3)cc1
Methylpropamine	N(C(Cc1sccc1)C)C
Amitriptyline	N(CC/C=C/1\c2c(cccc2)CCc2c\1cccc2)(C)C
Nortriptyline	N(CC/C=C/1\c2c(cccc2)CCc2c\1cccc2)C
Imipramine	N(CCCN1c2c(cccc2)CCc2c1cccc2)(C)C
Desipramine	N(CCCN1c2c(cccc2)CCc2c1cccc2)C
S-MDMA	N([C@@H](Cc1cc2OCOc2cc1)C)C
R-MDMA	N([C@H](Cc1cc2OCOc2cc1)C)C
Dimethylamylamine	NC(CC(CC)C)C
5-(2-aminopropyl)benzofuran (5-APB)	NC(Cc1cc2c(occ2)cc1)C
6-(2-aminopropyl)benzofuran (6-APB)	NC(Cc1cc2occc2cc1)C
Methylenedioxyaminoindane	NC1Cc2c(cc3OCOc3c2)C1
Methylenedioxyaminotetralin	NC1Cc2c(cc3OCOc3c2)CC1
S-Amphetamine	N[C@H](Cc1ccccc1)C
Nomifensine	Nc1c2c(C(c3ccccc3)CN(C)C2)ccc1
Nisoxetine	O(C(CCNC)c1ccccc1)c1c(OC)cccc1
GBR 12935	O(C(c1ccccc1)c1ccccc1)CCN1CCN(CCCc2ccccc2)CC1
1- Naphyrone	O=C(C(CCC)N1CCCC1)c1c2c(ccc1)cccc2
Naphyrone	O=C(C(CCC)N1CCCC1)c1cc2c(cc1)cccc2
Mephedrone	O=C(C(NC)C)c1ccc(C)cc1
Methylethcathinone	O=C(C(NCC)C)c1ccc(C)cc1
Methylenedioxy-N-benzulcathinone	O=C(C(NCc1ccccc1)C)c1cc2OCOc2cc1
Benzedrone	O=C(C(NCc1ccccc1)C)c1ccc(C)cc1
Cocaine	O=C(OC)[C@H]1[C@@H](OC(=O)c2ccccc2)C[C@H]2N(C)[C@@H]1CC2

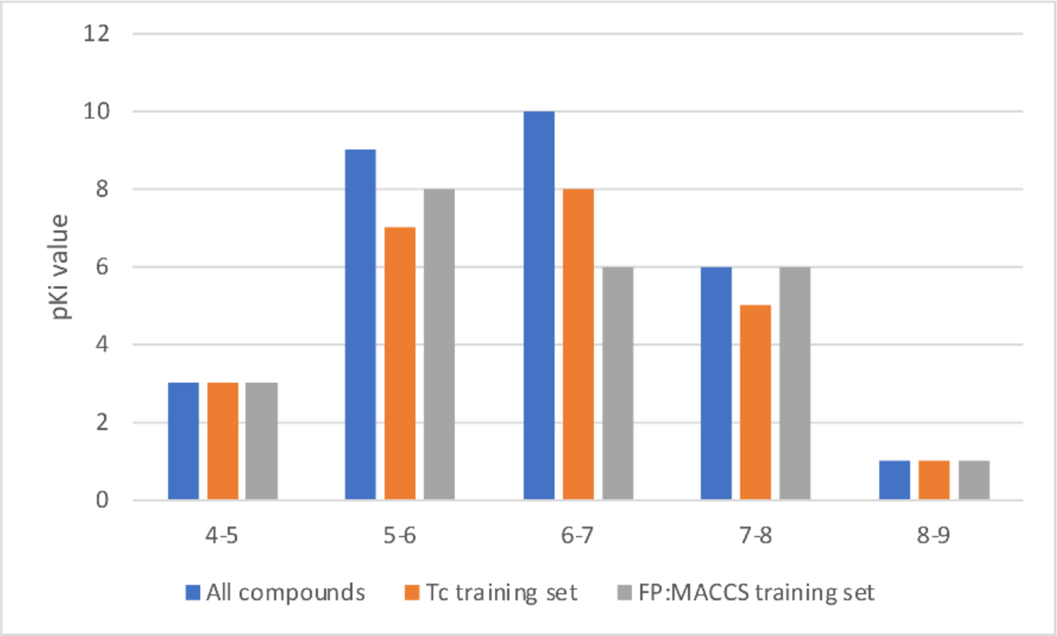

Figure S1. Distribution of compounds by pKi for DAT. Entire dataset (blue), Tanimoto-derived training set (orange) and FP: MACCS-derived training set (grey).

	1	2	3	4	5	6	7	8	9	10	11	12	13	14	15	16	17	18	19	20	21	22	23	24	25	26	27	28	29	30	31	Average
1	1	0.36	0.40	0.42	0.40	0.49	0.42	0.74	0.19	0.29	0.27	0.66	0.66	0.91	0.28	0.29	0.28	0.37	0.37	0.63	0.33	0.30	0.31	0.33	0.71	0.35	0.28	0.28	0.19	0.28	0.26	0.42
2	0.36	1	0.21	0.27	0.25	0.32	0.27	0.29	0.16	0.21	0.19	0.31	0.31	0.34	0.20	0.19	0.15	0.27	0.27	0.36	0.21	0.20	0.20	0.21	0.32	0.22	0.20	0.20	0.11	0.20	0.16	0.26
3	0.40	0.21	1	0.23	0.22	0.30	0.28	0.56	0.09	0.64	0.59	0.40	0.40	0.44	0.18	0.30	0.21	0.74	0.74	0.28	0.33	0.24	0.24	0.23	0.42	0.33	0.32	0.18	0.22	0.19	0.20	0.36
4	0.42	0.27	0.23	1	0.82	0.38	0.39	0.34	0.11	0.29	0.27	0.36	0.36	0.40	0.23	0.22	0.15	0.29	0.29	0.56	0.26	0.22	0.22	0.26	0.37	0.27	0.25	0.23	0.13	0.25	0.20	0.32
5	0.40	0.25	0.22	0.80	1	0.36	0.37	0.33	0.10	0.28	0.26	0.34	0.34	0.38	0.22	0.21	0.15	0.28	0.28	0.52	0.24	0.21	0.21	0.25	0.35	0.26	0.24	0.22	0.13	0.24	0.19	0.31
6	0.49	0.32	0.30	0.38	0.36	1	0.44	0.45	0.25	0.34	0.35	0.65	0.65	0.53	0.35	0.42	0.31	0.37	0.37	0.53	0.33	0.44	0.45	0.43	0.47	0.34	0.25	0.35	0.21	0.25	0.33	0.41
7	0.42	0.27	0.28	0.39	0.37	0.44	1	0.35	0.14	0.46	0.36	0.40	0.40	0.40	0.28	0.29	0.18	0.36	0.36	0.53	0.29	0.37	0.29	0.28	0.37	0.30	0.24	0.28	0.16	0.24	0.29	0.35
8	0.74	0.29	0.56	0.34	0.33	0.45	0.35	1	0.14	0.28	0.25	0.61	0.61	0.81	0.24	0.42	0.27	0.34	0.34	0.47	0.31	0.29	0.29	0.31	0.68	0.31	0.24	0.24	0.27	0.24	0.23	0.39
9	0.19	0.16	0.09	0.11	0.10	0.25	0.14	0.14	1	0.08	0.09	0.20	0.20	0.17	0.11	0.12	0.16	0.12	0.12	0.17	0.15	0.16	0.17	0.15	0.15	0.16	0.12	0.11	0.07	0.12	0.14	0.17
10	0.29	0.21	0.64	0.29	0.28	0.34	0.46	0.28	0.08	1	0.81	0.31	0.31	0.31	0.20	0.23	0.16	0.84	0.84	0.36	0.34	0.25	0.26	0.24	0.28	0.35	0.21	0.20	0.14	0.21	0.25	0.35
11	0.27	0.19	0.59	0.27	0.26	0.35	0.36	0.25	0.09	0.81	1	0.32	0.32	0.28	0.21	0.22	0.19	0.75	0.75	0.32	0.37	0.29	0.29	0.27	0.26	0.39	0.21	0.21	0.14	0.21	0.27	0.35
12	0.66	0.31	0.40	0.36	0.34	0.65	0.40	0.61	0.20	0.31	0.32	1	1.00	0.72	0.26	0.31	0.36	0.34	0.34	0.47	0.31	0.44	0.45	0.34	0.65	0.32	0.24	0.27	0.21	0.24	0.27	0.42
13	0.66	0.31	0.40	0.36	0.34	0.65	0.40	0.61	0.20	0.31	0.32	1.00	1	0.72	0.26	0.31	0.36	0.34	0.34	0.47	0.31	0.44	0.45	0.34	0.65	0.32	0.24	0.27	0.21	0.24	0.27	0.42
14	0.91	0.34	0.44	0.40	0.38	0.53	0.40	0.81	0.17	0.31	0.28	0.72	0.72	1	0.27	0.32	0.30	0.39	0.39	0.57	0.33	0.32	0.32	0.35	0.78	0.35	0.26	0.27	0.21	0.26	0.25	0.43
15	0.28	0.20	0.18	0.23	0.22	0.35	0.28	0.24	0.11	0.20	0.21	0.27	0.27	0.27	1	0.26	0.16	0.22	0.22	0.29	0.25	0.25	0.23	0.26	0.26	0.25	0.26	1.00	0.19	0.26	0.28	0.29
16	0.29	0.19	0.30	0.22	0.21	0.42	0.29	0.42	0.12	0.23	0.22	0.31	0.31	0.32	0.26	1	0.18	0.24	0.24	0.28	0.23	0.22	0.23	0.26	0.29	0.21	0.26	0.26	0.31	0.26	0.25	0.28
17	0.28	0.15	0.21	0.15	0.15	0.31	0.18	0.27	0.16	0.16	0.19	0.36	0.36	0.30	0.16	0.18	1	0.19	0.19	0.18	0.34	0.56	0.57	0.34	0.26	0.35	0.16	0.16	0.17	0.16	0.34	0.27
18	0.37	0.27	0.74	0.29	0.28	0.37	0.36	0.34	0.12	0.84	0.75	0.34	0.34	0.39	0.22	0.24	0.19	1	1.00	0.38	0.40	0.26	0.26	0.28	0.35	0.41	0.23	0.22	0.15	0.23	0.24	0.38
19	0.37	0.27	0.74	0.29	0.28	0.37	0.36	0.34	0.12	0.84	0.75	0.34	0.34	0.39	0.22	0.24	0.19	1.00	1	0.38	0.40	0.26	0.26	0.28	0.35	0.41	0.23	0.22	0.15	0.23	0.24	0.38
20	0.63	0.36	0.28	0.56	0.52	0.53	0.53	0.47	0.17	0.36	0.32	0.47	0.47	0.57	0.29	0.28	0.18	3.80	3.80	1	0.33	0.27	0.28	0.33	0.48	0.35	0.28	0.29	0.15	0.28	0.24	0.61
21	0.33	0.21	0.33	0.26	0.24	0.33	0.29	0.31	0.15	0.34	0.37	0.31	0.31	0.33	0.25	0.23	0.34	0.40	0.40	0.33	1	0.37	0.38	0.45	0.29	0.84	0.22	0.25	0.21	0.22	0.36	0.34
22	0.30	0.20	0.24	0.22	0.21	0.44	0.37	0.29	0.16	0.25	0.29	0.44	0.44	0.32	0.25	0.22	0.56	0.26	0.26	0.27	0.37	1	0.85	0.32	0.29	0.38	0.23	0.25	0.17	0.23	0.30	0.33
23	0.31	0.20	0.24	0.22	0.21	0.45	0.29	0.29	0.17	0.26	0.29	0.45	0.45	0.32	0.23	0.23	0.57	0.26	0.26	0.28	0.38	0.85	1	0.34	0.30	0.39	0.23	0.23	0.17	0.23	0.36	0.34
24	0.33	0.21	0.23	0.26	0.25	0.43	0.28	0.31	0.15	0.24	0.27	0.34	0.34	0.35	0.26	0.26	0.34	0.28	0.28	0.33	0.45	0.32	0.34	1	0.30	0.47	0.22	0.26	0.25	0.22	0.39	0.32
25	0.71	0.32	0.42	0.26	0.35	0.47	0.37	0.68	0.15	0.28	0.26	0.65	0.65	0.78	0.25	0.28	0.26	0.35	0.35	0.48	0.29	0.29	0.30	0.30	1	0.30	0.24	0.26	0.23	0.24	0.22	0.39
26	0.35	0.22	0.33	0.37	0.26	0.34	0.30	0.31	0.16	0.35	0.39	0.32	0.32	0.35	0.25	0.21	0.35	0.41	0.41	0.35	0.84	0.38	0.39	0.47	0.30	1	0.22	0.25	0.20	0.22	0.38	0.35
27	0.28	0.20	0.19	0.27	0.24	0.25	0.24	0.24	0.12	0.21	0.21	0.24	0.24	0.26	0.26	0.26	0.16	0.23	0.23	0.28	0.22	0.23	0.23	0.22	0.24	0.22	1	0.26	0.15	1.00	0.21	0.28
28	0.28	0.20	0.18	0.25	0.22	0.35	0.28	0.24	0.11	0.20	0.21	0.27	0.27	0.27	1.00	0.26	0.16	0.22	0.22	0.29	0.25	0.25	0.23	0.26	0.26	0.25	0.26	1	0.19	0.26	0.28	0.29
29	0.19	0.11	0.22	0.13	0.13	0.21	0.16	0.27	0.07	0.14	0.14	0.21	0.21	0.21	0.19	0.31	0.17	0.15	0.15	0.15	0.21	0.17	0.17	0.25	0.23	0.20	0.15	0.19	1	0.15	0.23	0.21
30	0.28	0.20	0.19	0.25	0.24	0.25	0.24	0.24	0.12	0.21	0.21	0.24	0.24	0.26	0.26	0.26	0.16	0.23	0.23	0.28	0.22	0.23	0.23	0.22	0.24	0.22	1.00	0.26	0.15	1	0.21	0.28
31	0.26	0.16	0.20	0.20	0.19	0.33	0.29	0.23	0.14	0.26	0.27	0.27	0.27	0.25	0.28	0.25	0.34	0.24	0.24	0.24	0.36	0.30	0.36	0.39	0.22	0.38	0.21	0.28	0.23	0.21	1	0.29

Figure S2: Correlation matrix (pairwise Tanimoto coefficients). Identical compounds would have a value of 1. Pairwise values below 0.20 are shown in red. The final column displays the average similarity coefficients for each compound. Compound 9 (dimethylamylamine) has an average Tc of 0.17 indicating it is structurally dissimilar to other compounds and should not be used in generation of QSAR models.

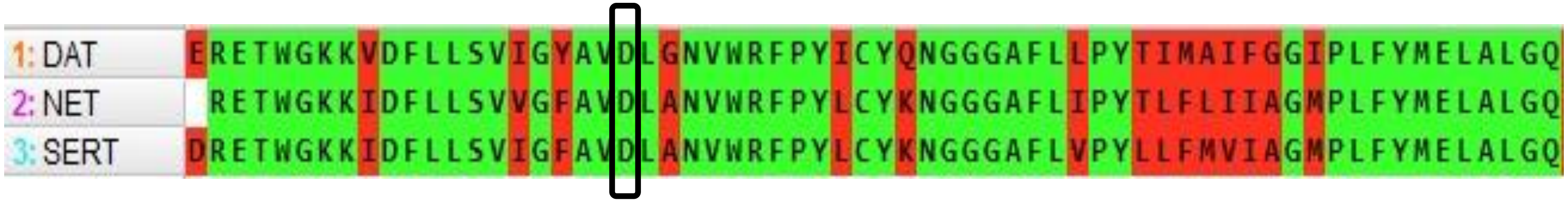

Figure S3: Partial amino acid sequence alignment for Q01959 (DAT), P23975 (NET) and P31645 (SERT) showing identical residues (green), non-identical residues (red) and highlighting the aspartate residue crucial to biogenic amine binding conserved across the isoforms (black box)

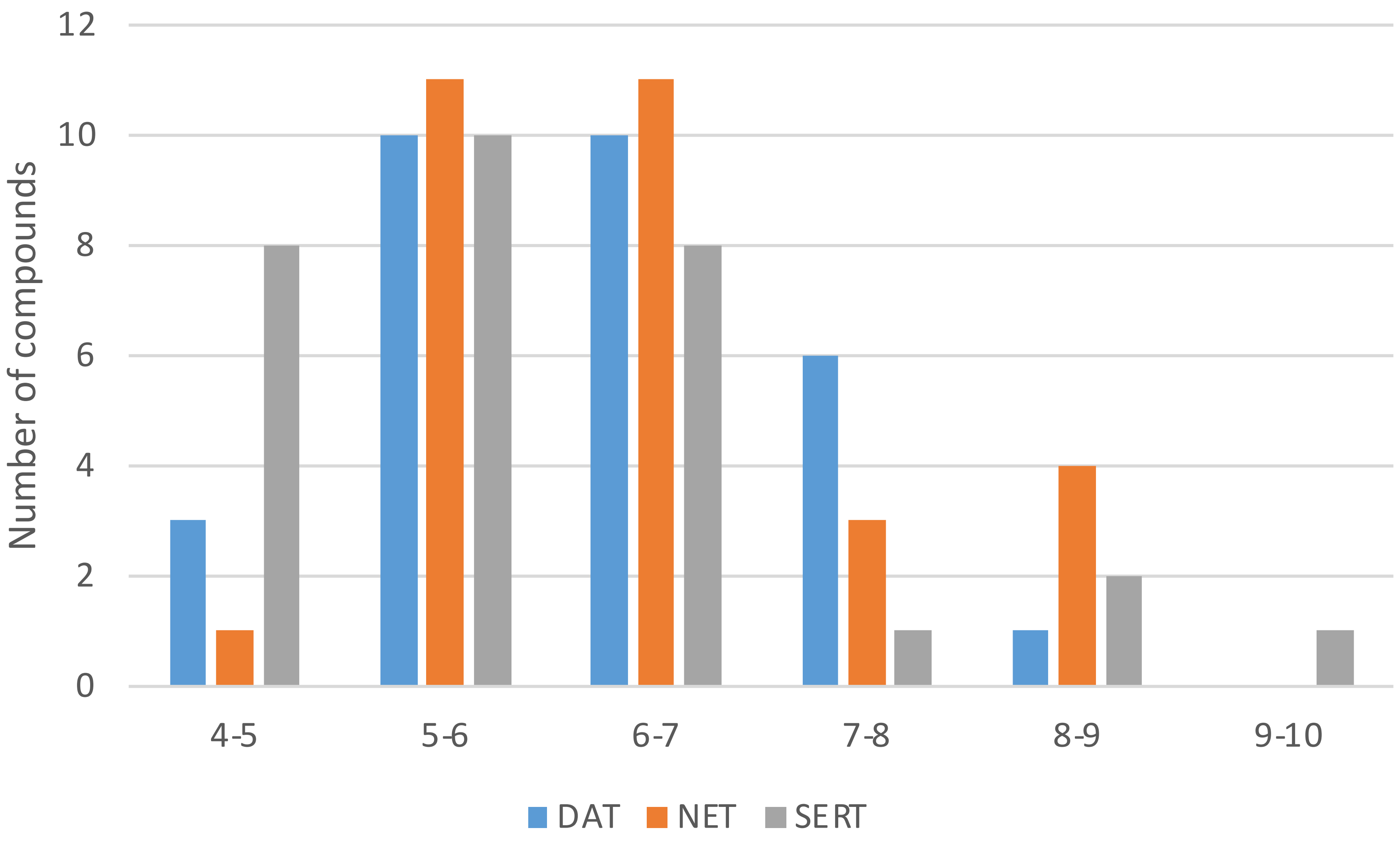

Figure S4: The relative distribution of experimental binding affinities, for DAT (blue), NET (orange) and SERT (grey).
